# Applying Behavioral and Physiological Measures to Assess the Relative Impact of the Prolonged COVID-19 Pandemic Closure on Two Mammal Species at the Oregon Zoo: Cheetah (*A. jubatus*) and Giraffe (*G. c. reticulata* and *G. c. tippelskirchii*)

**DOI:** 10.3390/ani11123526

**Published:** 2021-12-10

**Authors:** Laurel B. Fink, Candace D. Scarlata, Becca VanBeek, Todd E. Bodner, Nadja C. Wielebnowski

**Affiliations:** 1 Department of Biology, Portland State University, 1719 SW 10th Avenue, SRTC rm 246, Portland, OR 97201, USA; 2 Oregon Zoo, 4001 SW Canyon Rd, Portland, OR 97221, USA; candace.scarlata@oregonzoo.org (C.D.S.); becca_na@juno.com (B.V.); nadja.wielebnowski@oregonzoo.org (N.C.W.); 3 Department of Psychology, Portland State University, 1721 SW Broadway, Cramer Hall rm 317, Portland, OR 97201, USA; tbodner@pdx.edu

**Keywords:** visitor effect, zoo animal welfare, wildlife endocrinology, animal behavior

## Abstract

**Simple Summary:**

During the COVID-19 global pandemic the Oregon Zoo closed to all visitors and non-essential staff from 15 March 2020 to 12 July 2020. This presented a unique opportunity to conduct a study on the behavioral and physiological changes associated with the transitions between visitor presence and visitor absence on some of our more sensitive species. Using behavioral scan-sampling and fecal hormone monitoring of fecal glucocorticoid metabolites (fGM), two cheetahs and two giraffes were observed through two transition periods: the initial closure of the Oregon Zoo in March 2020 and the subsequent reopening in July 2020. We found significant increases in fGM concentrations for both cheetahs and giraffes between the two transition periods, but not within each specific transition. We also found some differences in behavior frequencies for both cheetahs and giraffes. For the cheetahs, ‘not visible’ significantly increased within the second transition period, resulting in their being less visible to visitors, and the cheetahs also spent less time showing locomotion behavior after the Zoo reopened. The giraffes, in turn, expressed significant differences in vigilant behaviors, with increased vigilance shown while fewer visitors were around. This study illustrates the complexity of investigating the visitor effect in different species and the individualistic nature of the responses of both the cheetahs and giraffes at the Oregon Zoo. Overall, however the observed changes in fGM concentrations and behavioral expressions were relatively minor and may have been more strongly related to social changes (giraffe) and medical events (cheetah) than to visitor activity and the Zoo’s opening status.

**Abstract:**

The effect of visitor presence on zoo animals has been explored in numerous studies over the past two decades. However, the opportunities for observations without visitors have been very limited at most institutions. In 2020, the Oregon Zoo was closed, in response to the global SARS-CoV-2 (COVID-19) pandemic, from 15 March 2020 to 12 July 2020, resulting in approximately four consecutive months without visitor presence. This study aimed to quantify potential behavioral and hormonal changes expressed during two transition periods in zoo visitor attendance: the initial time period before and after closure in March 2020 and time before and after reopening in July 2020. Fecal glucocorticoid metabolite (fGM) concentrations of resident giraffes (*n* = 2) and cheetahs (*n* = 2) were tracked using enzyme immunoassay (EIA) analyses. Average fGM concentrations during the two transition periods were compared using a two-way mixed ANOVA. Additionally, twice-weekly scan sampling was used to quantify behavioral observations across the transitions, which were analyzed as individual behavior proportions. Individual behavior proportions were compared across the Zoo’s opening status and time of day using Kruskal–Wallis (H) tests. The results of our analyses showed the following outcomes: (1) significant increases in fGM concentrations for cheetahs and giraffes between the transition periods but not within them; (2) a significant increase in time spent ‘not visible’ in the cheetahs in the second transition period; and (3) increased vigilance behaviors in the giraffes immediately after the Zoo’s closure. However, the changes observed in fGM concentrations may be more strongly correlated with concomitant social changes (giraffes) and some medical events (cheetahs) rather than with the Zoo’s opening status. Nevertheless, this study was able to quantify differences in behavioral frequencies and fGM concentration in cheetahs and giraffes at the Oregon Zoo during the times of transition between visitor’s presence and absence. The results indicate that, while there was a possible, but relatively minor impact of the presence and absence of visitors on some behaviors, the differences observed in fGM concentration may have been more affected by some of the concomitant social changes and medical events that happened during the same period than by the presence or absence of visitors.

## 1. Introduction

A longstanding interest in zoo researchers has been how the presence or absence of visitors affects the behavior and physiology of zoo animals. In 2000, Hosey [1] formally introduced the term ‘visitor effect’. Since then, this phenomenon has been studied in a wide variety of species. Typically, these studies have reported varying responses to visitor presence depending on individual and species-specific characteristics. For instance, increased visitor presence has been correlated with ‘less time visible to the public’ and increased vigilance (both frequently used to indicate a certain level of discomfort with visitor presence and, thus, possible reduced levels of well-being) for multiple species (orangutans—*Pongo pygmaeus* and *Pongo abelii* [2], koalas—*Phascolarctos cinereus* [3], ocelots—*Leopardus pardalis* and bobcats—*Lynx rufus* [4]). However, other studies have shown ambivalence or no reaction to visitor presence (see Davey [5] for a comprehensive review). Like humans, individuals within the same species can show different behavioral [6] and adrenal [7] responses to the same event. For instance, Razal et al. [8] reported significantly different mean response values for individual reticulated giraffe (*Giraffa camelopardalis*) behaviors between the same seasonal stressors and Polgár et al. [6] found differing behavioral responses to visitors by captive spider monkeys (*Saimiri sciureus*) based on personality traits. There are several different hypotheses as to why there are such variable responses to visitor presence, both between and within species, including differing individual traits (life histories, genetics, and temperaments) and life experiences as well as evolutionary background (see Sherwen and Hemsworth [9] for a review).

Visitors may impose three main influences on animals: visual, audible, and olfactory [10]. Recent studies have emphasized the importance of olfactory [11] and auditory [3,12,13,14] stressors when comparing visitor effects. Monitoring these stimuli can be complicated, with the two influences being almost impossible to isolate. One way to monitor the overall impact of the presence of visitors is to entirely eliminate them from the zoo during regular visitor hours and compare the results to normal periods. This is rarely possible due to the need for visitor associated revenue. Thus, a limitation of many visitor-effect studies to date has been the lack of data from times without any visitors. Such periods of complete visitor absence tend to be opportunistic and brief. For instance, Mallapur et al. [15] reported that captive lion-tailed macaques (*Macaca silenus*) showed a 20% decrease in short-term abnormal behaviors and a 30% decrease in long-term abnormal behaviors when visitors were absent. However, for the short-term study, the visitor absence period was limited to one day whereas the long-term study relied on a comparison between on-exhibit and off-exhibit behaviors. In other studies (e.g., Chiew et al. [16]), access to an exhibit was simply blocked off for a set period to reduce visitor presence. This strategy only highlights different behaviors related to visibility because the focal animals were still exposed to auditory and olfactory changes associated with nearby visitors.

Studies on physiological stress responses are now commonly conducted in combination with behavioral monitoring. Here, we define a stressor as any event that elicits an adrenal response, whether positive or negative. Many nondomestic animals may “hide” signs of stress, making it more difficult to recognize behavioral responses to stressors; yet such physiological reactions can be used as internal indicators of positive or negative stress responses when combined with other external indicators such as behaviors and/or animal health measures.

The activation of the hypothalamic-pituitary-adrenal (HPA) axis is one of the most consistent physiological responses to stimulation across different species and is involved regardless of whether the stressor is considered positive (e.g., mating) or negative (e.g., pain). One result of the HPA-axis stimulation is the eventual excretion of glucocorticoid metabolites, including cortisol and corticosterone, into the feces and/or urine, which can then be monitored to determine the intensity of the stressor (see O’Connor et al. [17] for a review of the HPA-axis). While it is possible to measure the concentration of intact glucocorticoids in plasma, the collection of plasma samples is inherently stressful and can influence the concentration of glucocorticoids in less than three minutes [18]. However, a substantial number of studies have shown that glucocorticoids, after being metabolized, get excreted in feces and urine, and the adrenal response to an intense stressor observed in serum samples can be measured in fecal or urine samples noninvasively [19,20,21,22,23].

Among available non-invasive techniques, fecal glucocorticoid metabolite (fGM) monitoring is the most popular for several reasons (see Palme [24] for a recent review). Fecal samples: (1) are most consistently available for noninvasive collection on wild and captive animals; (2) represent a pooled concentration of adrenal hormone levels over time (depending on species-specific metabolization and excretion patterns); and (3) may be less sensitive to diurnal fluctuations and short-term secretion of adrenal hormones into the bloodstream when compared to serum glucocorticoids [25]. As mentioned previously, the excretion of glucocorticoid metabolites occurs with differing instances of arousal and requires further interpretation (such as behavioral and/or animal health analyses) to determine if a given stressor is positively or negatively affecting an individual [26].

In 2020, the global SARS-CoV-2 (COVID-19) pandemic resulted in the temporary closure of most zoological institutions for periods ranging from weeks to months. On 15 March 2020, amid rapidly increasing positive COVID-19 cases, Oregon’s governor ordered the immediate closure of all large recreational facilities in the state. The subsequent closure of the Oregon Zoo resulted in nearly four consecutive months (until 12 July 2020) without visitors and with substantially reduced staffing. This extended closure provided researchers with the opportunity to quantify the potential behavioral changes expressed by some zoo animals that are typically subjected to high visitor attendance. Due to its urban location, the Oregon Zoo receives nearly 1.7 million people per year, with the most heavily attended months occurring in the summer [27]. Recent studies have quantified the effects of these closures in other institutions, primarily for mammals [28,29], but with at least one reptile study [30]. Williams et al. [29] showed a variety of behavioral changes in eight monitored species. Among these were an increase in ‘comfort’ behaviors (such as self-grooming or self-maintenance behaviors), closer presence to areas where visitors were usually located, and more environmental interactions (investigation or interaction of non-food items in the environment) during zoo closure periods. In contrast, Riley et al. [30] found significant differences in Nile crocodile (*Crocodylus niloticus*) behavior that more directly related to time of day, temperature, and month rather than the absence of visitors.

In addition to formal research, anecdotal stories reported by zoo staff indicated a range of different responses to the extended absence of visitors. For instance, Daniel Ashe (CEO of the Association of Zoos and Aquariums, AZA—Silver Spring, MD, USA), based on information received from a variety of AZA institutions and their animal care staff, reported “general boredom” across several species [31]. The same article reported that without visitors, normally “aloof” individuals such as gorillas (*Gorilla gorilla gorilla*) and camels (*Camelus bactrianus*) at the Calgary Zoo approached staff members more frequently for interaction. At the Phoenix Zoo, care staff used their breaks to interact with the petting zoo goats (*Capra hircus*) to counteract the dramatic decrease in human interaction to which goats were previously habituated. Even with such mitigation, the goats received less interaction than they were accustomed to during visitor times [32].

Here we report on a study aimed at quantifying how transitions in visitor attendance affected the physiology and behavior of animals at the Oregon Zoo. We tracked behavioral and adrenal responses to two transition periods in visitor absence (the initial closure and the reopening four months later), in giraffes (*Giraffa camelopardalis reticulata* and *Giraffa tippelskirchi*) and cheetahs (*Acinonyx jubatus*). We chose to focus our analyses on two transition periods, since they were deemed the times of most intense change. For each transition, we aimed to obtain comparable before and after data (equal numbers of observations and fecal samples).

We hypothesized that changes in fGM concentrations and behavior expression would be minimal during the first transition period and more variable during the second transition period, due to potential gradual acclimation to visitor absence over the months of closure and the sudden return of visitors. For behavior analyses, we hypothesized a transition to more exploratory activities and more ‘time spent visible’ without visitors, similarly to previously reported analyses [2,3,4].

We hypothesized that the effects of the initial shutdown would be less than the reopening transition because both species had previously experienced short-term periods without visitors. For both cheetahs and giraffes, these included inclement weather closures. Additionally, both cheetahs in this study had been recently housed in entirely “off-exhibit” habitats at their previous institution. As they only arrived in January 2020, they may have still been acclimated to limited visitor access. At the Oregon Zoo, both study species are popular with visitors, and their habitats are located along the main visitor pathway, providing a stark difference in visitor presence during the open and closed time periods. While previous studies have observed the visitor effect on both giraffes [33] and cheetahs [34], most giraffe behavioral studies have mainly centered around direct human-animal interactions, such as feeding platforms, rather than indirect visitor presence [33,35]. Many cheetah studies have focused on a single behavior (e.g., stereotypy [36]) or a specific relationship (e.g., mother-cub relationship [34]).

Since this study focused specifically on the transition periods in visitor attendance, it may provide valuable information about the effect of visitor presence on both species. If analyses reveal an increase or decrease in potential positive or negative indicators of stress, more in-depth studies would be needed to identify how the effects of visitor presence or absence can be mitigated appropriately.

## 2. Materials and Methods

All research was reviewed and approved by the Oregon Zoo Research Review Committee (OZRRC).

### 2.1. General

The Oregon Zoo is located just outside the downtown area of Portland, Oregon, USA. We collected data on two visitor presence transition periods. Each transition period consisted of two month-long treatments, one with visitors and one without. Transition Period 1 included the initial opening period of full visitor access (“Open”) and the first period of complete visitor absence (“Closed A”). Transition Period 2 included the end of the visitor absence period (“Closed B”) and the beginning of visitor access period (“Reopen”). However, visitor access was somewhat variable between the two transition periods. During “Open” there were no restrictions on visitor numbers, in both “Closed (A)” and “Closed (B)” visitors were unable to attend the Zoo, and in “Reopen” there was a 50% visitor capacity cap. Data collection specific to this study took place between mid-March 2020 and August 2020; however, since both species were part of the Zoo’s ongoing welfare monitoring program (including behavioral and physiological monitoring), some of the previously collected data between January 2020 and early March 2020 were included in the analyses. We decided to focus on the data directly associated with the two main transition periods to provide comparable ‘before and after’ data for statistical analyses as well as to isolate specific changes associated with the transition times.

### 2.2. Focal Animals

#### 2.2.1. Cheetah (A. jubatus)

We monitored two 10-year-old male cheetahs (“B10183” and “B10184”) that had recently (January 2020) returned to the Oregon Zoo after spending three years at the San Diego Wild Animal Park (SD-WAP). Both cheetahs were provided a diet consisting of feline-specific chow (Milliken Carnivore) and game carcasses. Fresh water was available ad libitum. The cheetah habitat included three indoor off-exhibit areas (one 109 ft^2^ and two 80 ft^2^ habitats) and a large grassy outdoor exhibit (approximately 19,200 ft^2^) viewable by the public. The outdoor cheetah yard consisted of a small shelter with heated flooring on the western side, a small pond on the eastern, and a large grassy knoll in the middle which prevents visitor viewing to the back of the exhibit. The cheetahs participated in voluntary training sessions four to five times per week (average duration 5 min). The training durations and frequencies remained consistent over the course of the shutdown. Both cheetahs had pre-existing health issues prior to arriving at the Oregon Zoo, and several medical procedures were conducted between 6 April 2020 and 6 July 2020.

#### 2.2.2. Giraffe (*G.c. reticulata* and *G. tippelskirchi*)

For this study, we observed two male giraffes, a reticulated giraffe (“B20186”, 9 years old, *G.c. reticulata*) and a Masai giraffe (“B60180”, 5 years old, *G. tippelskirchi*). In late May 2020 a young female Masai giraffe (“C00028”, 2 years old) was introduced to the herd but was not included in the study. According to fecal progesterone metabolite tracking, the giraffe C00028 was not cycling throughout the duration of this study. Additionally, there was minimal difference in analyzed fGM concentrations following giraffe C00028′s introduction between the two males, despite differences in fecundity (giraffe B20186 was castrated in 2012), which indicated no significant changes in fGM concentrations based on androgen production. The two males had arrived at the Oregon Zoo in 2012 and 2016, respectively. All giraffes shared a multi-species exhibit along the main Zoo pathway with three southern ground hornbills (*Bucorvus leadbeateri*) and one Speke′s gazelle (*Gazella spekei*). All giraffes were fed a combination of alfalfa and pellets (Mazuri Wild Herbivore Hi-Fiber Cube 5V05) daily. Browse was hung around the exhibit while carrot and primate L/S biscuits (cinnamon, Mazuri 5M1S) were supplemented in training sessions when available. Fresh water was available ad libitum. The giraffe habitat included an indoor barn for protection in inclement weather and two large outdoor areas (combined approximate area is 20,000 ft^s^) which could be separated by a fence, if necessary. The outdoor areas were covered with dirt, decomposed granite substrate, and grass. There were multiple logs (enrichment items for the Speke’s gazelle and the southern ground hornbills), a large pond, and multiple large trees scattered throughout the environment which provided shade, browse, and enrichment. The indoor barn consisted of multiple stalls with a rubber-coated concrete flooring lightly covered in wood shavings and was maintained at 65 °F (18.3 °C). Visitor viewing access was provided through raised walkways along the southern edge of the outdoor exhibit. The giraffe exhibit also included a feeding platform; however, it was not yet in regular use at the time the COVID-19 closure began and remained closed to the public.

During the day, the giraffes were housed solely indoors when temperatures fell below 40 °F (4.44 °C) but they were allowed outdoors for a maximum of four hours if temperatures were between 40 °F and 50 °F (10 °C) with no precipitation. If temperatures were >50 °F, the giraffes were given continuous access to the outdoor area (except when the outdoor exhibit was actively being maintained by care staff). To maintain a safe environment and minimize slipping hazards, the giraffes were housed indoors whenever there was ice or snow present on exhibit, or if there was significant rainfall. The temperatures remained adequate for outdoor housing throughout the entire study time and all behavior observations occurred outdoors.

### 2.3. Endocrinology

#### 2.3.1. Sample Collection

As previous research has indicated, fGM concentrations change significantly following transfers [37,38,39], introductions [40,41], and veterinary procedures [42]. Sample dates were chosen to minimize the effects of these additional stressors while still providing meaningful data on transition periods.

Fecal samples were collected three times per week between 07:00 and 10:00 by animal care staff during the two transition periods (Table 1). Care staff were instructed to avoid samples contaminated by urine or other substances. Samples were labelled with name, species, date, and time collected and immediately frozen (−4 °F/−20 °C) until analysis. Food-grade dye was used to identify samples from individuals housed together.

#### 2.3.2. Steroid Extraction

Fecal glucocorticoid metabolites (fGM) were extracted using an adaptation of methods documented by Bryant and Wielebnowski [43]. Briefly, 0.500 g (±0.025 g) of wet, homogenized fecal matter was mixed with 5 mL 80% alcohol (giraffe: methanol; cheetah: ethanol), vortexed, and shaken overnight for 17 h (Fisherbrand^TM^ open air rocking shaker). Then, the samples were vortexed again and centrifuged (Sorvall^TM^ ST 16) at 2500 rpm for 15 min. Three milliliters of the resulting 1:1 supernatant was removed and 500 µL was subsequently desiccated in a SpeedVac (Savant Speedvac DNA110) before being reconstituted in 500 µL Tris HCl assay buffer. Using the results of in-house parallelism analyses, dilutions were then created with Tris assay buffer based on optimum concentrations for the detection of fGMs.

#### 2.3.3. Enzyme Immunoassay (EIA) Analysis

It has been previously determined that glucocorticoid metabolites can be reliably extracted from fecal samples in both target species (giraffe [44], cheetah [45]). A double-antibody corticosterone enzyme immunoassay (EIA) (CJM006, 1:100,000, C. Munro, University of California, Davis, CA, USA) was used for both species. A 96-well microtiter plate was pre-coated with secondary goat anti-rabbit IgG antibody (150 µL/well at 0/10 mg/mL, A009, Arbor Assays, Ann Arbor, MI, USA) using the standard Arbor Assay methodology. The inter- and intra-assay coefficients of variation (CV) were maintained below 15% and 10%, respectively. The corticosterone antibody cross-reacts at 100% with corticosterone, 14.25% with desoxycorticosterone, 2.65% with progesterone, 0.90% with tetrahydrocorticosterone, 0.64% with testosterone, 0.23% with cortisol, and less than 0.10% for five other steroids tested (C. Munro).

**Figure 1:**
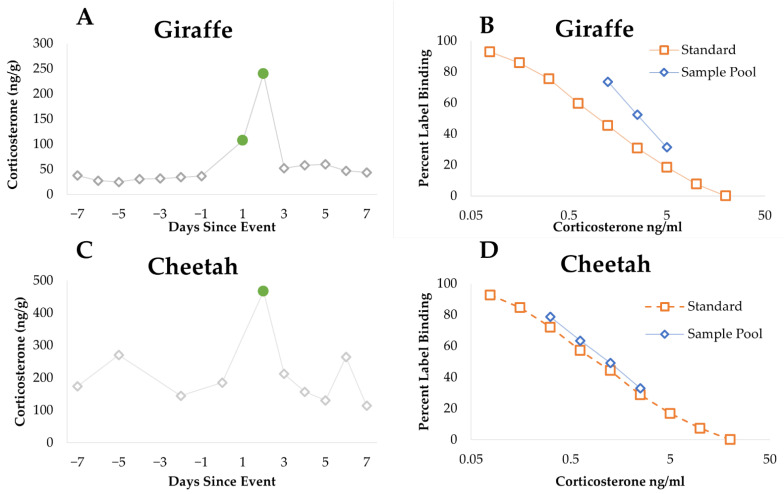
Endocrinology validation data for giraffes and cheetahs. (**A**) Biological validation of giraffes displaying significant peaks post-institution transfer. (**B**) Parallelism for giraffes of pooled samples and standards using antibody “CJM006”. (**C**) Biological validation of cheetahs displaying a significant peak post-surgery. (**D**) Parallelism for cheetahs of pooled samples and standards using antibody “CJM006”. Highlighted (●) data points are >2 standard deviations above the calculated baseline for each individual.

The corticosterone EIA was validated by showing: (1) parallelism between binding inhibition curves of a corticosterone standard curve and a serially diluted pool of fecal extractions for each species, (2) a biological validation for each species due to a significant increase (less than two standard deviations over baseline) in fGM concentrations within 48 h of an invasive veterinary procedure (cheetahs) or an exhibit transfer (giraffes) (Figure 1), and (3) no significant cross-reactivity with fGM concentrations and androgen concentrations.

#### 2.3.4. Data Analysis

Analyses were conducted using IBM SPSS Statistics for Windows (version 21.0). A repeated measure mixed two-way ANOVA model was used to determine potential differences in average fGM concentrations for each treatment. Shapiro–Wilks tests of normality revealed non-normal datasets for both the cheetah and giraffe fGM data. Once log-transformed, all data were normally distributed except for data included in B10184: Reopen (*p* = 0.037). However, due to the small sample size and the robustness of the ANOVA tests, this break in assumption was ignored. Comparison of studentized residuals indicated no extreme outliers (no studentized residuals exceeded ± 3) for either species. Levene’s test of homogeneity (*p* > 0.05) and Box’s M test (*p* > 0.001) revealed homogenous variances and covariances, respectively. For cheetahs, Mauchly’s test of Sphericity indicated a violation, χ^2^ = 14.253, *p* = 0.014, and the Huynh–Feldt epsilon adjustment was used to circumnavigate this violation. Neither cheetahs, (*F*(2.584, 72.349) = 9.937, *p* = 0.136, partial η^2^ = 0.065, ε = 0.861 (Huynh-Feldt adjustment)), nor giraffes, (*F*(3,84) = 0.934, partial η^2^ = 0.032, *p* = 0.428) had significant differences between individuals and were, therefore, analyzed within species-specific groups. All data is reported after having been back-transformed, unless otherwise stated.

### 2.4. Behavior

Previously developed ethograms used at the Oregon Zoo for ongoing welfare monitoring were replicated for this project ([46,47]: Table 2 and Table 3). One-minute scan sampling occurred over 20-min observation periods twice per day (1000 and 1430) two days a week (see Table 4 for observation dates). Due to the strong likelihood of care staff presence influencing behavior, any observation during which a member of the care staff was present was omitted from the analysis. Each species was observed from the general visitor viewing areas. Due to restrictions from COVID-19 safety protocols, uneven observation sampling was inevitable. Behavioral observations for the category “Open” were conducted through the general welfare monitoring program at the Oregon Zoo prior to the commencement of the study and were 60-min observation periods, rather than 20-min observation periods, conducted by the first author for the rest of the study. It is important to note that the 60-min observations were conducted by volunteers, rather than the main researcher. However, each volunteer was required to pass a reliability test in order to collect behavior data (behavior observation reliability >80% compared with the author’s observations using procedures from Wark et al. [48]). We therefore felt that these observations could be included in the overall analyses. To make the data comparable over the different observation durations, all count data were converted into percent of behavioral expression using the following equation:

$$\%\;Behavior\;Expression=\frac{Sum\;of\;specific\;behavior\;count\;data}{Sum\;of\;cumulative\;observation\;period}\times 100$$

#### Data Analysis

Analyses were conducted using IBM SPSS Statistics for Windows (version 21.0). Two behavior analyses were conducted per focal animal, comparisons of percent of time spent engaging in each behavior across zoo opening status and time of day. For both species, Shapiro–Wilks tests and visual observations of QQ plots revealed non-normal (*p* < 0.05) data despite transformations. Therefore, non-parametric Kruskal–Wallis tests were conducted on the original, untransformed data. For both species, distributions of behavior proportions were not similarly shaped across each opening status or time of day, as assessed by visual inspections of boxplots. Data are reported as differences in mean ranks of behavior proportions by zoo opening status or time of day. Each individual focal animal was analyzed separately.

## 3. Results

### 3.1. Endocrinology

#### 3.1.1. Cheetah

**Figure 2:**
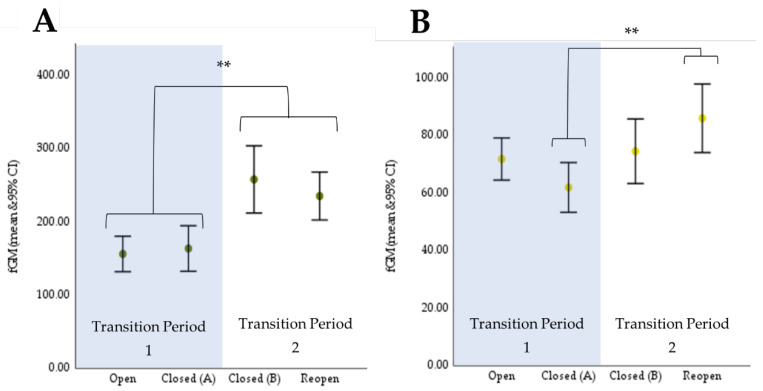
Results from repeated measures mixed 2-way ANOVA models. Asterisks (**) indicate significance at α = 0.90. Data are back transformed from log-transformed data. Error bars are 95% CI. (**A**) Average cheetah fGM concentrations. (**B**) Average giraffe fGM concentrations.

There was a significant increase in fGM concentrations between Transition Period 1 and Transition Period 2. There was no significant difference between the samples collected within each transition period. The repeated measures mixed two-way ANOVA model indicated that these differences in fGM concentrations were statistically significant based on zoo opening status, (*F*(2.584, 72.349) = 9.937, *p* < 0.001, partial η^2^ = 0.262 (Huynh-Feldt adjustment)) (Table 5, Figure 2A).

#### 3.1.2. Giraffe

There were statistically significant differences in fGM concentrations between the different treatments, (*F*(3,84) = 4.154, *p* = 0.009, partial η^2^ = 0.129). Similar to cheetahs, there was no statistically significant difference between the samples collected within each treatment period. However, there was a statistically significant increase in average fGM concentration of 22.7 ng/g (*p* = 0.027) between samples collected during “Closed (A)” and during “Reopen”. This indicates a significant difference in average fGM concentration between the end of Transition Period 1 and the end of Transition Period 2 (Table 5, Figure 2B).

### 3.2. Behavior

#### 3.2.1. Cheetah

**Figure 3:**
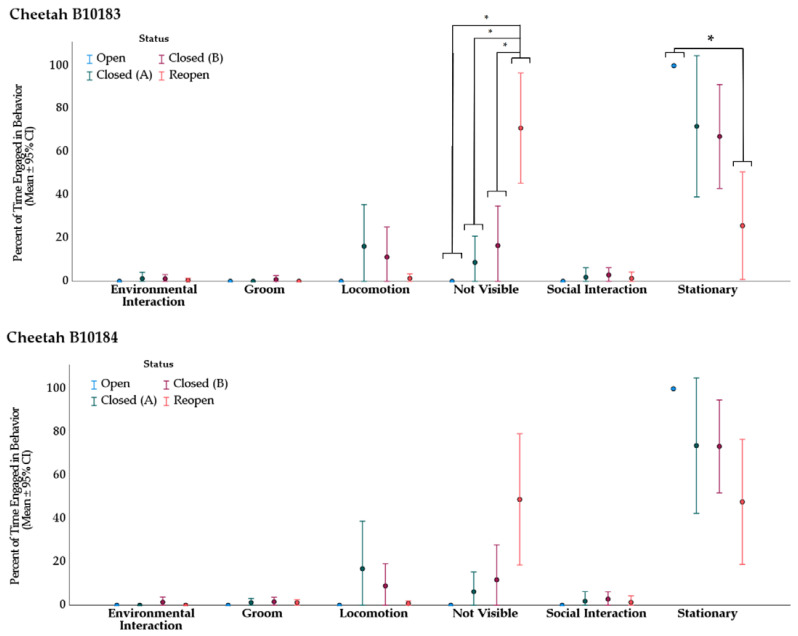
Cheetah average behavior expression proportion (%). Proportion indicates percent of total observation time conducting the behavior. Error bars are 95% confidence intervals. Asterisks (*): significance at α = 0.95. Observations in category “Open” were 60-minutes and conducted by volunteers. All others were 20-minutes and conducted by the first author.

The Kruskal–Wallis (H) tests revealed two significant changes in behavior frequencies for cheetah B10183 and no significant differences in behavior frequencies for cheetah B10184, based on the Zoo’s opening status (Figure 3). The mean ranks of cheetah B10183′s behavior frequencies were statistically different between the Zoo’s opening status for “not visible”, χ^2^(3) = 15.07, *p =* 0.002, and “stationary”, χ^2^(3) = 10.81, *p* = 0.013. Subsequently, pairwise comparisons (Dunn 1964, Bonferroni correction for multiple comparisons) were conducted (Table 6).

#### 3.2.2. Giraffe

**Figure 4:**
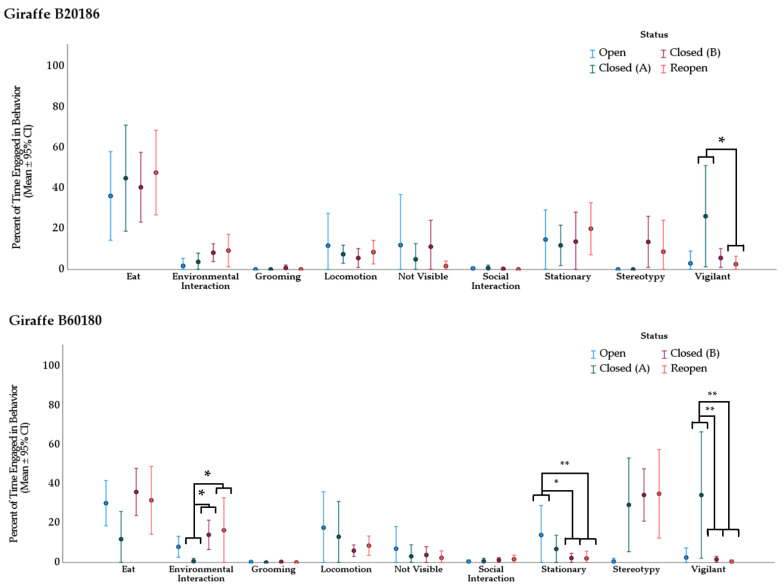
Giraffe average behavior expression proportion (%). Proportion indicates percent of total observation time conducting the behavior. Error bars are 95% confidence intervals. Asterisks (*): significance at α = 0.95, (**): significance at α = 0.90. Observations in category “Open” were 60-minutes and conducted by volunteers. All others were 20-minutes and conducted by the first author.

Kruskal–Wallis (H) tests revealed one difference in behavior frequency for giraffe B20186 and three significant changes in behavior frequency for giraffe B60180 based on zoo opening status (Figure 4). For giraffe B20186, there was a significant difference in behavior frequency for “vigilant”, χ^2^(3) = 8.378, *p* = 0.039. For giraffe B60180 significant differences in behavior frequencies were present for “environmental interaction”, χ^2^(3) = 10.688, *p* = 0.014; “stationary”, χ^2^(3) = 12.761, *p* = 0.005; and “vigilant”, χ^2^(3) = 17.826, *p* < 0.001).

Subsequent pairwise comparisons (Dunn 1964) were conducted on each of the significant results. *p*-values are reported with Bonferroni corrections to account for multiple comparisons (Table 7).

## 4. Discussion

This study took advantage of a unique opportunity of prolonged closure of the Oregon Zoo due to the COVID-19 pandemic to provide a novel contribution to the body of research on the impact of visitor presence on two sensitive yet popular species. Our initial hypotheses were: (1) fGM concentrations would decrease in periods without visitors, but this effect would be smaller in the first transition period than the second one, due to potential acclimation to a lack of visitors over four months of closure; and (2) there would be a transition to more exploratory behaviors and less time spent hidden without visitors. We found that the first hypothesis was partially supported by giraffe fGM concentrations, and the second hypothesis was partially supported by the behavior data obtained for both species. The results were more complex than expected.

For the two male cheetahs, significant increases in average fGM concentrations were observed between the two transition periods, but not within each transition period. The fGM concentrations of treatments “Open” and “Closed (A)” were both significantly lower than the fGM concentrations of treatments “Closed (B)” and “Reopen”, While the overall average fGM concentration increased, the differences within the actual transitions were minimal. This indicates that there may have been some different factors (aside from visitor presence or absence) that were underlying the observed significant increases in fGM concentrations. A study by Uetake et al. [45] revealed a trending decrease in cheetah fGM concentrations within the summer months and high fGM concentrations on the day after a strong decrease in air temperature—hypothesizing sensitivity to cold temperatures. This is contradictory to that revealed in our study—indicating that it may not be simply seasonal fluctuations causing the increased fGM concentrations in cheetahs at the end of the summer. However, both cheetahs were subject to more frequent veterinary exams and treatments than usual during the study period. Research has shown that increased veterinary care and repeated veterinary procedures can lead to increased glucocorticoid secretion, in spite of the overall beneficial effects for the individual [50]. While we attempted to minimize the effect of veterinary exams on the study by choosing data samples that avoided major procedures, previous injuries and some dental issues observed in the cheetahs resulted in three veterinary procedures for cheetah B10184 and one for cheetah B10183 between April 2020 and August 2020. The effects of these exams may have had a substantial and lingering effect on fGM concentrations during the study period. These medical procedures were, most likely, influential factors on the significant difference in fGM concentrations between the two transition periods and may have overshadowed differences in fGM concentrations based on visitor presence. Without these medical procedures, it is possible that the cheetahs would have better followed patterns found by previous researchers that indicate significant increases in fGM concentrations in cheetahs housed on-exhibit verses off-exhibit [51]. Several of the procedures were considered to be relatively minor in terms of invasiveness of medical procedures, yet stress responses are individualistic in nature and depend on the individual animal’s history and perception of the event. However, there was one surgical procedure for cheetah B10184 on 14 June 2020. In addition, both cheetahs historically had issues with mutual reintroductions after veterinary procedures and this also may have affected their respective fGM concentrations—regardless of which cheetah experienced the veterinary procedure.

Similarly, the samples collected for the giraffe analyses also showed minimal differences within transition periods, but significant differences between the different transition periods. While periods without visitors were found to be lower in fGM concentrations than the samples associated with visitor presence, these differences were statistically insignificant. However, the trending increase in fGM concentrations within the reopening transition period was similar to other visitor presence studies on ungulates (Indian blackbuck—*Antilope cervicapra* [52], chamois—*Rupicapra rupicara* [53]). Again, the sample dates were chosen to avoid intense stressors that may obscure a possible effect of the visitor transition periods. In May 2020, a two-year-old female Masai giraffe (C00028) was added to the herd. While giraffe C00028 did not show active progesterone cycles during the study, the addition of a female to our all-male herd caused social changes that may have influenced the fGM concentrations of both male giraffes during and at the end of the summer close to the second transition period. Additionally, research has shown an increase in adrenal hormone concentrations for wild African ungulates during the dry season [54], similar to the patterns of fGM concentration changes observed during this study. In Portland, Oregon, the dry season stretches from mid-May to mid-October [55]. Our male giraffes had significantly higher fGM concentrations in the samples collected in July than March, which followed the seasonality patterns seen in other ungulates.

Individuals of both species experienced significant changes that were social in nature for the giraffes and medical for the cheetahs between the Zoo’s closure and reopening periods that may explain at least some portion of the increasing fGM concentrations for the second transition period. However, the Zoo’s opening status may still have added to the overall effect of increased fGM concentrations observed after reopening the Zoo. It is possible that the absence of visitors temporarily prevented a larger compounding effect of visitor presence and social/health impacts in each species.

Nevertheless, comparisons of individual behavior frequencies resulted in multiple significant differences for each species; however, none of these significant differences occurred within a transition period. For instance, the giraffes showed significant increases in vigilance, environmental interaction, and significant decreases in stationary behaviors, while one cheetah showed a significant increase in time spent ‘not visible’ and decreased stationary behaviors.

The giraffes were found to be more vigilant when the Zoo was initially closed, however, this difference was statistically trending. This slight change may have been due to the sudden lack of foot traffic associated with the closure between “Open” and “Closed (A)”. The results indicated possible acclimation to the lack of visitors, with both giraffe B60180 and giraffe B20186 showing less vigilant behaviors between “Closed (A)” and “Closed (B)”. When the Zoo is open, visitors tend to move quickly and consistently through the area, possibly causing something akin to a ‘white noise’ effect, and individual spectators may not be noticed much by the giraffes. However, when the Zoo is closed, the giraffes may pay more attention to single individuals (such as care staff or security) on the observation decks. Previous research supports this hypothesis by showing an increased rate of vigilant behaviors in ungulates with decreasing human activity [13]. Additionally, research conducted on various zebra exhibits found that alert (in some papers, alert and vigilance share the same definition) and locomotive behaviors tended to decrease as visitor numbers increased [56]. Interestingly, a recent study showed a positive correlation in the time the giraffes spent ‘observing the observer’ (author definition of vigilance behavior [57], page six) and number of observers present. However, this study had a maximum of three observers—drastically less than the typical visitor numbers experienced in a zoo setting—and the quantity of observers may not have reached the ‘white noise’ effect. Another potential explanation for the increased vigilance behavior is the possibility of food begging. Prior to the closure, both male giraffes were being conditioned to hand feeding from visitors located on a feeding platform. It is possible that the giraffes were looking for the observer to provide food. This follows the pattern discussed by Lynn [58], who theorized that the presence of visitors on a platform may increase attentiveness (similar behavior to ‘vigilance’ in our study) of giraffes due to the giraffes’ association of visitor presence and food. However, our study varies from this previous research in that vigilance increased with decreasing visitor presence. This indicates that the difference in vigilance we observed may be a combination of food-begging and the ability for the giraffes to focus on a single visitor rather than the ‘white noise’ of the popular visitation periods. There were other significant differences in behavior for each giraffe, but they were also between different transitional periods and most likely related to other stressors outside visitor presence. For instance, the significant increases between environmental interactions in “Closed (A)” and both “Closed (B)” and “Reopen” for giraffe B60180 may be partially explained by the changes in season. The earliest leaf budding (lilacs and honeysuckle) was reported in early March 2020 [59] and the giraffes may have been searching for new buds. As our ethogram only categorizes eating behavior as observable feeding, foraging behaviors that did not result in successful eating were considered as environmental interactions. Nevertheless, the changes are most likely associated with the significant increase in vigilant behaviors. As we only score one behavior per scan, if an individual is expressing one behavior it means they are not expressing another. For “Closed (A)”, there was a dramatic increase in vigilant behaviors that directly resulted in a loss of expression of all other behaviors. This is common when using scan-sampling techniques and necessitates the conversion of behavior occurrences into proportions prior to data analyses [60]. Additionally, there were differences in overnight housing due to low temperatures in the first transition period. While all observations were conducted when the giraffes were in their outdoor habitat, it is possible that the changes in overnight housing may have affected the behavior frequencies and require further investigation.

For the cheetahs, the increased time spent ‘not visible’ was the most notable response to the effect of visitor absence. Unlike the differences in the giraffes’ behavior expressions, there was a significant increase in time spent ‘not visible’ within the second transitional period. This indicates that visitor presence may have a stronger influence on this behavioral difference than other stressors. Frequently, an animal’s increased avoidance of visitor viewing areas is used as an indication of negative visitor effects [61,62,63]. For the cheetahs at the Oregon Zoo, observing such a strong increase in time spent ‘not visible’ may indicate they had acclimated to the prolonged time of visitor absence. We note that there was not a decrease in time spent ‘not visible’ when the Zoo was first closed, which would have been a strong indication that visitor presence directly influenced cheetah exhibit use. However, this may be attributed to the presence of a heated area located at the front of the exhibit in which the cheetahs frequently nap during the colder months. This explanation is also supported by the inverse relationship between time spent ‘not visible’ and stationary behaviors with the least time spent ‘not visible’ corresponding with the most stationary behaviors. These findings support the hypothesis that the cheetahs at the Oregon Zoo acclimated to the long period without visitors, but it also indicates that the time of year and various environmental factors may have a strong influence on behavior responses.

Additionally, evolutionary backgrounds of either species may have contributed to the differing behavior expressions. A comprehensive study by Queiroz and Young [64] provided empirical evidence for behavioral and evolutionary traits that could predict which species may be the most sensitive to visitor presence. The researchers found that herbivorous, terrestrial, diurnal species from historically closed habitats would likely be the most vulnerable to visitor presence [64]. Giraffes are an herbivorous, terrestrial species that follows a diurnal activity pattern, albeit mostly found in more open habitat. According to their evolutionary history, giraffes may, therefore, be somewhat more susceptible to behavior changes based on visitor presence compared with other non-herbivorous species. Cheetahs, however, are crepuscular or diurnal terrestrial predators. Based on Queiroz and Young’s predictive traits, the cheetahs should be less susceptible to visitor presence than giraffes. However, among medium-to-large predators, cheetahs are known to be more timid and to usually avoid confrontation. From an evolutionary perspective, cheetahs mostly need to outrun their predators and competitors, making them also potentially more vulnerable to visitor impact than other same-size or larger predators. Previous research has also shown that wild cheetahs choose environments with greater numbers of large predators (e.g., lions and leopards) over environments with human settlements and may view humans as a larger threat [65]. Additional research conducted by Terio et al. [66], indicating significantly higher fGM concentrations in captive cheetahs than free-ranging cheetahs, supports this idea. In many zoos, including the Oregon Zoo, cheetahs are housed in exhibits with combined ‘off-exhibit’ (no visitor viewing) and ‘on-exhibit’ (visitor viewing) access. However, to maximize the visitor viewing experience, many times cheetahs are only allowed access to their off-exhibit areas at night. This can result in limited retreat options for cheetahs and force cheetahs to remain in an environment that prevents the expression of their natural hiding and escape behaviors, possibly affecting changes in behavior expression (e.g., more hiding, less restful behaviors) and increased fGM concentrations. This was reported by Baird [51], who found significantly higher fGM concentrations in cheetahs housed on-exhibit than those housed off-exhibit. In summary, it is essential to fully understand a species’ natural history and behaviors when designing visitor accessibility to minimize the potential negative effects of visitor attendance.

While this study revealed some significant changes in behavior and minimal changes in fGM concentrations associated with transitions in visitor attendance, we acknowledge the limitations of the study. Since behavioral data for the “Open” observation period were collected through general welfare monitoring sessions (prior to the official start of the study) rather than in conjecture with a formal study, observations did not occur as frequently as for the other categories. These data, while limited in frequency, were important to include as they do represent behaviors observed for both species within the time frame for baseline data observation, while visitors were present under pre-COVID conditions. Second, only two individuals per species were analyzed, since these represented the available study population for the species at Oregon Zoo at the time when the study started. Third, behavior expression and fGM concentration reactions are unique to both species and to the individuals within each species. Multiple individuals of the same species can be exposed to the exact same stressor but show differing physiological and behavioral responses, depending on genetics, life experience, gender, and age.

Finally, the effect of each transition period appeared to be relatively minor for each of the species and individuals. This study highlights that visitor presence is less influential than other factors, such as veterinary procedures, social changes, and seasonal fluctuations. This is an important conclusion; visitor presence is a factor in almost all zoo animals’ environment. While visitor presence may be a low-grade chronic stressor, it is not as influential as other factors the animals experience. It is, therefore, of major importance to carefully document and consider all life events possibly impacting an individual’s experience when analyzing data obtained for the purpose of visitor presence studies.

## 5. Conclusions

While the global COVID-19 pandemic has had many negative impacts on zoos and aquaria, especially from a financial perspective, it did allow for an unusual study opportunity of zoo animals without any visitors present. This has allowed us to gain more insight into which behaviors cheetahs and giraffes at the Oregon Zoo may exhibit when only in contact with care staff. The current study was an opportunistic attempt to document behavior and hormonal changes observed during periods of transition in visitor access in two mammal species we had been already monitoring.

The results of this study indicate two important conclusions: (1) there were significant differences in the adrenal activity of giraffes and cheetahs at the Oregon Zoo between transition periods but not within them and (2) there was only one significant difference in behavior frequency (cheetah: ‘not visible’) that occurred within a transition period. All other significant behavioral changes were between the two different visitor transition periods. This indicates visitor presence may less prominently affect behavior an fGM concentrations on zoo-housed cheetahs and giraffes than other factors, such as seasonal fluctuations, social changes, or medical procedures.

While we note the limitations of this study (small sample size, uneven observation counts, and possible compounding effects), we believe that some of the results may warrant further examination. The finding of an increased amount of time spent ‘not visible’ for cheetahs when the Oregon Zoo reopened may indicate a need for additional retreat areas to provide choices for cheetah to move further away from the public and have a comfortable space to do so, when needed. For giraffe, the increased vigilance behavior may indicate a heightened sensitivity to small amounts of visitors or small groups rather than large consistently moving groups and this phenomenon might benefit from further studies, to look at the impact of small visitor group access afterhours or for giraffe feeding.

Finally, we reiterate the individualistic nature of both behavioral and adrenal responses to stressors and to emphasize the difficulty in crediting these changes to a single cause given the various changes that occurred concomitantly with changes in visitor presence.

## Figures and Tables

**Table 1:** List of different treatment periods, their corresponding dates and the number of fecal samples collected within the time. Samples analyzed within Open and Closed (A) are part of Transition Period 1 while samples analyzed in Closed (B) and Reopen are part of Transition Period 2.

Cheetah				
	Category	Sample Dates	Sample Count (B10183)	Sample Count (B10184)
Transition Period 1	Open	2/20/2020–3/15/2020	15	15
	Closed (A)	3/16/2020–4/4/2020	15	15
Transition Period 2	Closed (B)	6/16/2020–7/12/2020	15	15
	Reopen	7/13/2020–8/7/2020	15	15
**Giraffe**				
	**Category**	**Sample Dates**	**Sample Count (B20186)**	**Sample Count (B60180)**
Transition Period 1	Open	2/13/2020–3/15/2020	15	15
	Closed (A)	3/16/2020–4/12/2020	15	15
Transition Period 2	Closed (B)	6/9/2020–7/12/2020	15	15
	Reopen	7/13/2020–8/8/2020	15	15

**Table 2:** Pre-established cheetah ethogram [46].

All-Occurrence Behavior	Description
charge glass	Charge towards glass ending within one body length of the glass; may or may not include a strike or hiss
glass strike	Forceful paw contact with glass
hiss	Lips pulled back to bare teeth and emit sound
**Interval Behaviors**	**Description**
not visible	Individual out of sight or unable to determine behavior at interval
keeper visible	Keeper is present—can be actively interacting with focal animal or just walking past
environmental interaction	Individual is actively engaged with an element of its environment; does not include interaction with zoo visitors or inactive contact with environment (e.g., laying down on rocks) or incidental contact with exhibit furniture
stereotypy	Locomotor stereotypy: walking from one point to another, turning and walking back to the starting point, or walking in a loop/to-and-fro, for more than three repetitions without interruption.
social interaction	Any active social interaction with another cheetah, regardless of who instigated it
locomotion	Any movement that transports the animal more than one body length forward, backward, or sideways at any speed; includes walk, trot, run or jump
groom	Focal animal is engaged in self-grooming, licking, chewing, scratching (self)
stationary	Not deliberately exhibiting locomotion behaviors for at least three seconds; can be alert (head up, eyes open) and resting (head down or head up with closed eyes)

**Table 3:** Pre-established giraffe ethogram [47].

All-Occurrence Behavior	Description
urine testing	Using flehmen reaction specifically at urine
flehmen	Upper lip curled back and inhalation
interaction with Speke’s gazelle	Any interaction between giraffes and resident Speke’s gazelle
interaction with hornbills	Any interactions between giraffes and resident hornbills
run	Cantering or sprinting
lay down	Any instance when the giraffe has its stomach on the ground
**Interval Behaviors**	**Description**
not visible	Individual out of sight or unable to determine behavior at interval
keeper visible	Keeper is present—can be actively interacting with focal animal or just walking past
eat	Individual is actively eating from designated food stations or keeper-provided browse elements
environmental interaction	Individual is actively engaged with an element of its environment; does not include interaction with zoo visitors or inactive contact with the environment (e.g., laying down on rocks) or incidental contact with exhibit furniture.
stereotypy	Locomotor stereotypy: walking from one point to another, turning and walking back to the starting point, or walking in a loop/to-and-fro, for more than three repetitions without interruption; non-locomotor stereotypy: repetitive licking/tongue flagging
social interaction	Any active social interaction with another giraffe, regardless of who instigated it
locomotion	Any movement that transports the animal more than one body length forward, backward, or sideways at any speed; includes walk, trot, run or jump
groom	Focal animal is engaged in self-grooming, licking, chewing, scratching (self)
vigilant	Standing still with an erect neck and actively observing (rather than scanning) the environment (similar to that defined by Cameron and du Toit [49])
stationary	Not deliberately exhibiting locomotion behaviors for at least three seconds; can be alert (head up, eyes open) and resting (head down or head up with closed eyes)

**Table 4:** List of treatments and their corresponding dates and observation counts. Uneven observation counts are due to restrictions from COVID-19 safety protocols. Asterisks (*) indicate observations from general monitoring (60 min observations instead of 20 min observations) and conducted by volunteers. ^A^: indicates variable observation dates due to the lack of exhibit access because of routine habitat maintenance.

Cheetah				
	Category	Sample Dates	Observation Count (B10183)	Observation Count (B10184)
Transition Period 1	Open *	2/11/2020–3/15/2020	3	2
	Closed (A)	3/16/2020–4/4/2020 ^A^	8	8
Transition Period 2	Closed (B)	6/16/2020–7/12/2020 ^A^	14	14
	Reopen	7/13/2020–8/8/2020	13	13
**Giraffe**				
	**Category**	**Sample Dates**	**Observation Count (B20186)**	**Observation Count (B60180)**
Transition Period 1	Open *	1/18/2020–3/15/2020	4	4
	Closed (A)	3/16/2020–4/12/2020 ^A^	8	8
Transition Period 2	Closed (B)	6/9/2020–7/12/2020 ^A^	16	16
	Reopen	7/13/2020–8/8/2020	12	12

**Table 5:** Results of cheetah and giraffe fGM concentration analysis. Repeated measures two-way mixed ANOVA model comparing different zoo opening statuses. The mean difference results compare fGM concentrations in (I) trials with those in (II) trials. Asterisks (*) indicate significant (*p* < 0.05) differences between trial (I) and trial (II). ^a^ Bonferroni correction for multiple comparisons.

	(I) Trial	(II) Trial	Mean Difference (A-B) (ng/g)	Sig ^a^
Cheetah				
Transition Period 1	Open	Closed (A)	−6.06	1.00
		Closed (B) *	−87.46 *	0.008 *
		Reopen *	−79.63 *	<0.001 *
	Closed (A)	Open	6.06	1.00
		Closed (B) * Reopen *	−81.40 * −73.56 *	<0.001 *0.002 *
Transition Period 2	Closed (B)	Open *	87.46 *	0.008 *
		Closed (A) *	81.40 *	<0.001 *
		Reopen	7.83	1.00
	Reopen	Open *	79.63 *	<0.001 *
		Closed (A) *	73.56 *	0.002 *
		Closed (B)	−7.83	1.00
**Giraffe**				
Transition Period 1	Open	Closed (A)	11.13	0.053
		Closed (B)	0.00	1.000
		Reopen	−11.57	1.000
	Closed (A)	Open	−11.13	0.053
		Closed (B)	−11.13	0.262
		Reopen	−22.70 *	0.016 *
Transition Period 2	Closed (B)	Open	0.00	1.000
		Closed (A)	11.13	0.262
		Reopen	−11.57	1.000
	Reopen	Open	11.57	1.000
		Closed (A)	22.70 *	0.016 *
		Closed (B)	11.57	1.000

**Table 6:** Significant (α = 0.95) results of the behavior engagement analysis for cheetah based on the Zoo’s opening status. Pairwise comparisons (Dunn 1984) were conducted on significant results from Kruskal–Wallis (H) tests. Asterisks (*) indicate adjusted *p*-values (Bonferroni correction for multiple comparisons).

Cheetah B10183						
Behavior	Treatment (I)	Treatment (II)	Mean Rank (I)	Mean Rank (II)	Direction of Change	Adj. *p*-Value *
not visible	Open	Reopen	10.00	28.27	↑	0.034
	Closed (A)	Reopen	15.25	28.27	↑	0.030
	Closed (B)	Reopen	15.82	28.27	↑	0.010
stationary	Open	Reopen	30.50	12.31	↓	0.043

**Table 7:** Significant (α = 0.95) results of the behavior engagement analysis for giraffe based on zoo opening status. Pairwise comparisons (Dunn 1984) were conducted on significant results from Kruskal–Wallis (H) tests. Asterisks (*) indicate adjusted *p*-values (Bonferroni correction for multiple comparisons).

Giraffe B20186						
Behavior	Treatment (I)	Treatment (II)	Mean Rank (I)	Mean Rank (II)	Direction of Change	Adj. *p*-Value *
vigilant	Closed (A)	Reopen	29.63	15.79	↓	0.039
**Giraffe B60180**						
environmental interaction	Closed (A)	Closed (B)	8.81	23.97	↑	0.013
	Closed (A)	Reopen	8.81	23.42	↑	0.030
stationary	Open	Closed (B)	34.88	17.81	↓	0.016
	Open	Reopen	34.88	16.29	↓	0.009
vigilant	Closed (A)	Closed (B)	32.81	17.59	↓	0.003
	Closed (A)	Reopen	32.81	14.67	↓	0.001

## Data Availability

The data presented in this study are available on request from the corresponding author. The data are not publicly available due to the confidential nature of health data at the Oregon Zoo.

## References

[1] Hosey G.R. (2000). Zoo Animals and Their Human Audiences: What Is the Visitor Effect?. Anim. Welf..

[2] Choo Y., Todd P.A., Li D. (2011). Visitor effects on zoo orangutans in two novel, naturalistic enclosures. Appl. Anim. Behav. Sci..

[3] Larsen M.J., Sherwen S.L., Rault J.L. (2014). Number of nearby visitors and noise level affect vigilance in captive koalas. Appl. Anim. Behav. Sci..

[4] Suárez P., Recuerda P., Arias-De-Reyna L. (2017). Behaviour and welfare: The visitor effect in captive felids. Anim. Welf..

[5] Davey G. (2007). Visitors’ Effects on the Welfare of Animals in the Zoo: A Review. J. Appl. Anim. Welf. Sci..

[6] Polgár Z., Wood L., Haskell M.J. (2017). Individual differences in zoo-housed squirrel monkeys’ (*Saimiri sciureus*) reactions to visitors, research participation, and personality ratings. Am. J. Primatol..

[7] Wolf T.E., Bennett N.C., Burroughs R., Ganswindt A. (2018). The impact of age-class and social context on fecal glucocorticoid metabolite levels in free-ranging male giraffes. Gen. Comp. Endocrinol..

[8] Razal C., Bryant J., Miller L. (2017). Monitoring the behavioral and adrenal activity of giraffe (*Giraffa camelopardalis*) to assess welfare during seasonal housing changes. Anim. Behav. Cogn..

[9] Sherwen S.L., Hemsworth P.H. (2019). The Visitor Effect on Zoo Animals: Implications and Opportunities for Zoo Animal Welfare. Animals.

[10] Young R.J. (2003). Housing. Environmental Enrichment for Captive Animals.

[11] Farrand A. (2007). The Effect of Zoo Visitors on the Behaviour and Welfare of Zoo Mammals.

[12] Robbins L., Margulis S.W. (2014). The effects of auditory enrichment on gorillas. Zoo Biol..

[13] Brown C.L., Hardy A.R., Barber J.R., Fristrup K.M., Crooks K.R., Angeloni L.M. (2012). The effect of human activities and their associated noise on ungulate behavior. PLoS ONE.

[14] Quadros S., Goulart V.D.L., Passos L., Vecci M.A.M., Young R.J. (2014). Zoo visitor effect on mammal behaviour: Does noise matter?. Appl. Anim. Behav. Sci..

[15] Mallapur A., Sinha A., Waran N. (2005). Influence of visitor presence on the behaviour of captive lion-tailed macaques (*Macaca silenus*) housed in Indian zoos. Appl. Anim. Behav. Sci..

[16] Chiew S.J., Butler K.L., Fanson K.V., Eyre S., Coleman G.J., Sherwen S.L., Melfi V., Hemsworth P.H. (2021). Effects of the presence of zoo visitors on zoo-housed little penguins (*Eudyptula minor*). N. Z. J. Zool..

[17] O’Connor T.M., O’Halloran D.J., Shanahan F. (2000). The stress response and the hypothalamic-pituitary-adrenal axis: From molecule to melancholia. QJM-Mon. J. Assoc. Physicians.

[18] Romero L.M., Reed J.M. (2005). Collecting baseline corticosterone samples in the field: Is under 3 min good enough?. Comp. Biochem. Physiol. Mol. Integr. Physiol..

[19] Stead S.K., Meltzer D.G.A., Palme R. (2012). The measurement of glucocorticoid concentrations in the serum and faeces of captive African elephants (*Loxodonta africana*) after ACTH stimulation: Research communication. J. S. Afr. Vet. Assoc..

[20] Sheriff M.J., Krebs C.J., Boonstra R. (2010). Assessing stress in animal populations: Do fecal and plasma glucocorticoids tell the same story?. Gen. Comp. Endocrinol..

[21] Sheriff M.J., Dantzer B., Delehanty B., Palme R., Boonstra R. (2011). Measuring stress in wildlife: Techniques for quantifying glucocorticoids. Oecologia.

[22] Touma C., Palme R. (2005). Measuring Fecal Glucocorticoid Metabolites in Mammals and Birds: The Importance of Validation. Ann. N. Y. Acad. Sci.

[23] Möstl E., Palme R. (2002). Hormones as indicators of stress. Domest. Anim. Endocrinol..

[24] Palme R. (2019). Non-invasive measurement of glucocorticoids: Advances and problems. Physiol. Behav..

[25] Wielebnowski N., Watters J. (2007). Applying Fecal Endocrine Monitoring to Conservation and Behavior Studies of Wild Mammals: Important Considerations and Preliminary Tests. Isr. J. Ecol. Evol..

[26] Wielebnowski N. (2003). Stress and distress: Evaluating their impact for the well-being of zoo animals. J. Am. Vet. Med. Assoc..

[27] The Oregon Zoo Oregon Zoo Foundation 2010–2011 Gratitude Report. https://www.oregonzoo.org/about/about-oregon-zoo.

[28] Williams E., Carter A., Rendle J., Ward S.J. (2021). Understanding impacts of zoo visitors: Quantifying behavioural changes of two popular zoo species during COVID-19 closures. Appl. Anim. Behav. Sci..

[29] Williams E., Carter A., Rendle J., Ward S.J. (2021). Impacts of COVID-19 on Animals in Zoos: A Longitudinal Multi-Species Analysis. J. Zool. Bot. Gard..

[30] Riley A., Terry M., Freeman H., Alba A.C., Soltis J., Leeds A. (2021). Evaluating the Effect of Visitor Presence on Nile Crocodile (*Crocodylus niloticus*) Behavior. J. Zool. Bot. Gard..

[31] Wright R. Some Zoos, and Some of Their Animals, May Not Survive the Pandemic. https://www.newyorker.com/news/our-columnists/some-zoos-and-some-of-their-animals-may-not-survive-the-pandemic.

[32] Frank B.J. Phoenix Zoo Working to Combat Loneliness Felt by Animals. https://www.azcentral.com/story/news/local/phoenix/2020/04/04/phoenix-zoo-working-combat-loneliness-felt-animals-during-coronavirus/2948477001/.

[33] Normando S., Pollastri I., Florio D., Ferrante L., Macchi E., Isaja V., de Mori B. (2018). Assessing animal welfare in animal-visitor interactions in zoos and other facilities. A pilot study involving giraffes. Animals.

[34] Donovan D.O., Hindle J.E., Mckeown S., Donovan O. (1993). Effect of visitors on the behaviour of female Cheetahs and cubs. Int. Zoo Yearb..

[35] Orban D.A., Siegford J.M., Snider R.J. (2016). Effects of guest feeding programs on captive giraffe behavior. Zoo Biol..

[36] Quirke T., O’riordan R.M., Zuur A. (2012). Factors influencing the prevalence of stereotypical behaviour in captive cheetahs (*Acinonyx jubatus*). Appl. Anim. Behav. Sci..

[37] Fanson K.V., Lynch M., Vogelnest L., Miller G., Keeley T. (2013). Response to long-distance relocation in Asian elephants (*Elephas maximus*): Monitoring adrenocortical activity via serum, urine, and feces. Eur. J. Wildl. Res..

[38] Grandin T. (1997). Assessment of Stress during Handling and Transport. J. Anim. Sci..

[39] Volfová M., Machovcová Z., Schwarzenberger F., Voslářová E., Bedáňová I., Večerek V. (2019). The effects of transport stress on the behaviour and adrenocortical activity of the black-and-white ruffed lemur (*Varecia variegata*). Acta Vet. Brno.

[40] Fazio J.M., Freeman E.W., Bauer E., Rockwood L., Brown J.L., Hope K., Siegal-Willott J., Parsons E.C.M. (2020). Longitudinal fecal hormone monitoring of adrenocortical function in zoo housed fishing cats (*Prionailurus viverrinus*) during institutional transfers and breeding introductions. PLoS ONE.

[41] Loeding E., Thomas J., Bernier D., Santymire R. (2011). Using Fecal Hormonal and Behavioral Analyses to Evaluate the Introduction of Two Sable Antelope at Lincoln Park Zoo. J. Appl. Anim. Welf. Sci..

[42] Rothschild D.M., Serfass T.L., Seddon W.L., Hegde L., Fritz R.S. (2008). Using Fecal Glucocorticoids to Assess Stress Levels in Captive River Otters. J. Wildl. Manag..

[43] Bryant J., Wielebnowski N.C. (2018). Environmental impact on activity level and fecal glucocorticoid metabolite concentration of African elephants and black rhinoceros at brookfield zoo. Int. Int. J. Avian Wildl. Biol..

[44] Bashaw M.J., Sicks F., Palme R., Schwarzenberger F., Tordiffe A.S.W., Ganswindt A. (2016). Non-invasive assessment of adrenocortical activity as a measure of stress in giraffe (*Giraffa camelopardalis*). BMC Vet. Res..

[45] Uetake K., Une Y., Ito S., Yamabe M., Toyoda H., Tanaka T. (2014). Relationship of climatic conditions to fecal corticosterone levels of captive cheetahs reared in Japan. Anim. Sci. J..

[46] Lewis K., Wielebnowski N. (2020). (Oregon Zoo, OR, USA) Oregon Zoo General Monitoring Ethogram—Cheetah.

[47] Lewis K., Wielebnowski N. (2020). (Oregon Zoo, OR, USA) Oregon Zoo General Monitoring Ethogram—Giraffe.

[48] Wark J.D., Wierzal N.K., Cronin K.A. (2021). Gaps in Live Inter-Observer Reliability Testing of Animal Behavior: A Retrospective Analysis and Path Forward. J. Zool. Bot. Gard..

[49] Cameron E.Z., du Toit J.T. (2005). Social influences on vigilance behaviour in giraffes, *Giraffa camelopardalis*. Anim. Behav..

[50] Vonderen I.K., Kooistra H.S., Rijnberk A. (1998). Influence of Veterinary Care on the Urinary Corticoid: Creatinine Ratio in Dogs. J. Vet. Intern. Med..

[51] Baird B.A. (2018). Ambassador Animal Welfare: Using Behavioral and Physiological Indicators to Assess the Well-Being of Animals Used for Education Programs in Zoos.

[52] Rajagopal T., Archunan G., Sekar M. (2011). Impact of Zoo Visitors on the Fecal Cortisol Levels and Behavior of an Endangered Species: Indian Blackbuck (*Antelope cervicapra* L.). J. Appl. Anim. Welf. Sci..

[53] Zwijacz-Kozica T., Selva N., Barja I., Silván G., Martínez-Fernández L., Illera J.C., Jodłowski M. (2012). Concentration of fecal cortisol metabolites in chamois in relation to tourist pressure in Tatra National Park (South Poland). Acta Theriol. (Warsz.).

[54] Cizauskas C.A., Turner W.C., Pitts N., Getz W.M. (2015). Seasonal Patterns of Hormones, Macroparasites, and Microparasites in Wild African Ungulates: The Interplay among Stress, Reproduction, and Disease. PLoS ONE.

[55] Average Weather in Portland, Oregon, United States, Year Round-Weather Spark. https://weatherspark.com/y/757/Average-Weather-in-Portland-Oregon-United-States-Year-Round.

[56] Conte C. (2014). Do Visitors Affect Zebra Behavior in Zoos?. Biol. Sci..

[57] Scheijen C.P.J., van der Merwe S., Ganswindt A., Deacon F. (2021). Anthropogenic influences on distance traveled and vigilance behavior and stress-related endocrine correlates in free-roaming giraffes. Animals.

[58] Lynn B.L. (2018). Zoo Giraffe Welfare: A Literature Review and the Behavioral Effects of Guest Feeding Programs.

[59] (2020). USA National Phenology Network Historical Annual Spring Indices (2016-Previous Year), First Leaf-Spring Index. https://data.usanpn.org/geoserver-request-builder?service=wms&layer=si-x:average_leaf_ncep_historic&year=2020&format=image/jpeg&projection=4269&width=1700&height=800&state_border=1.

[60] Martin P., Bateson P. (2007). Measuring Behavior: An Introductory Guide.

[61] Ross S.R., Schapiro S.J., Hau J., Lukas K.E. (2009). Space use as an indicator of enclosure appropriateness: A novel measure of captive animal welfare. Appl. Anim. Behav. Sci..

[62] Sherwen S.L., Harvey T.J., Magrath M.J.L., Butler K.L., Fanson K.V., Hemsworth P.H. (2015). Effects of visual contact with zoo visitors on black-capped capuchin welfare. Appl. Anim. Behav. Sci..

[63] Sherwen S.L., Magrath M.J.L., Butler K.L., Hemsworth P.H. (2015). Little penguins, *Eudyptula minor*, show increased avoidance, aggression and vigilance in response to zoo visitors. Appl. Anim. Behav. Sci..

[64] Queiroz M.B., Young R.J. (2018). The different physical and behavioural characteristics of zoo mammals that influence their response to visitors. Animals.

[65] Klaassen B., Broekhuis F. (2018). Living on the edge: Multiscale habitat selection by cheetahs in a human-wildlife landscape. Ecol. Evol..

[66] Terio K.A., Marker L., Munson L. (2004). Evidence for chronic stress in captive but not free-ranging cheetahs (Acinonyx jubatus) based on adrenal morphology and function. J. Wildl. Dis..

